# Impact of humidity on aerosol growth from methanesulfonic acid

**DOI:** 10.1039/d5ea00123d

**Published:** 2026-07-06

**Authors:** Wenjuan Yu, Rima Baalbaki, Jiali Shen, Jenna DeVivo, Lucía Caudillo-Plath, Eva Sommer, Arto Heitto, Hannah Klebach, Douglas M. Russell, António Amorim, Hannah Beckmann, Nirvan Bhattacharyya, Vine Blankenship, Anouck Chassaing, Romulo Cruz-Simbron, Lubna Dada, Aenne Jacobshagen, Bernhard Judmaier, Milin Kaniyodical Sebastian, Ruth Konrat, Timm Krüger, Felix Kunkler, Clara J. Lietzke, Lu Liu, Roy Mauldin, Bernhard Mentler, Aleksandra Morawiec, Pedro Rato, Birte Rörup, Samuel Ruhl, Wiebke Scholz, Mario Simon, Alexandria Stinchfield, António Tomé, Yandong Tong, Jens Top, Nsikanabasi Silas Umo, Gabriela R. Unfer, Lejish Vettikkat, Jakob Weissbacher, Christos Xenofontos, Boxing Yang, Marcel Zauner-Wieczorek, Jiangyi Zhang, Zhensen Zheng, Theodoros Christoudias, Joachim Curtius, Imad El Haddad, Richard Flagan, Armin Hansel, Hartwig Harder, Andreas Kürten, Ottmar Möhler, Tuukka Petäjä, Rainer Volkamer, Paul M. Winkler, Douglas R. Worsnop, Markku Kulmala, Neil M. Donahue, Jasper Kirkby, Taina Yli-Juuti, Ilona Riipinen, Xu-Cheng He, Katrianne Lehtipalo

**Affiliations:** a Institute for Atmospheric and Earth System Research/Physics, Faculty of Science, University of Helsinki 00014 Helsinki Finland xucheng.he@helsinki.fi; b Climate and Atmosphere Research Center, The Cyprus Institute 1645 Nicosia Cyprus; c Helsinki Institute of Physics, University of Helsinki Helsinki Finland; d Center for Atmospheric Particle Studies, Department of Chemistry, Carnegie Mellon University Pittsburgh PA USA; e Institute for Atmospheric and Environmental Sciences, Goethe University Frankfurt Altenhoeferallee 1 60438 Frankfurt am Main Germany; f CERN, European Organisation for Nuclear Research 1211 Geneva Switzerland; g Faculty of Physics, University of Vienna Boltzmanngasse 5 1090 Wien Austria; h Department of Technical Physics, University of Eastern Finland P. O. Box 1627 70211 Kuopio Finland; i Faculdade de Ciências da Universidade de Lisboa and LIP Edificio C8, Campo Grande 1749-016 Lisboa Portugal; j Faculty of Science and Technology, University of Tartu 50411 Tartu Estonia; k Division of Chemistry and Chemical Engineering, California Institute of Technology 1200 E California Blvd Pasadena CA 91125 USA; l Department of Environmental Science, Stockholm University Stockholm Sweden; m Bolin Centre for Climate Research, Stockholm University Stockholm Sweden; n Department of Chemistry & Cooperative Institute for Research in Environmental Sciences (CIRES), University of Colorado Boulder Boulder CO 80309 USA; o PSI Center for Energy and Environmental Sciences, Paul Scherrer Institute 5232 Villigen PSI Switzerland; p Institute of Ion Physics and Applied Physics, University of Innsbruck Technikerstraße 25 6020 Innsbruck Austria; q Institute of Meteorology and Climate Research Atmospheric Aerosol Research, Karlsruhe Institute of Technology Karlsruhe Germany; r Atmospheric Chemistry Department, Max Planck Institute for Chemistry 55128 Mainz Germany; s Department of Atmospheric and Oceanic Sciences, University of Colorado Boulder CO USA; t IDL-Universidade da Beira Interior Rua Marquês D'Ávila e Bolama 6201-001 Covilhã Portugal; u Department of Chemistry and Biochemistry, University of North Carolina Wilmington Wilmington North Carolina 28403 USA; v Atmospheric Microphysics Department, Leibniz Institute for Tropospheric Research Leipzig Germany; w IONICON Analytik GmbH 6020 Innsbruck Austria; x Aerodyne Research Billerica MA USA; y Aerodyne Research, Joint International Research Laboratory of Atmospheric and Earth System Sciences, School of Atmospheric Sciences, Nanjing University 210023 Nanjing China; z Aerosol and Haze Laboratory, Beijing Advanced Innovation Center for Soft Matter Science and Engineering, Beijing University of Chemical Technology 100029 Beijing China; a Finnish Meteorological Institute Helsinki Finland

## Abstract

Methanesulfonic acid (MSA; CH_3_SO_3_H), produced by oxidation of dimethylsulfide (DMS; CH_3_SCH_3_), is a key precursor for aerosols in the marine boundary layer and free troposphere. Laboratory experiments show that MSA contributes to both particle nucleation and subsequent growth, often together with sulfuric acid (H_2_SO_4_) and base vapours such as ammonia (NH_3_). However, the influence of relative humidity (RH) on MSA condensation to particles remains uncertain. Here, in experiments conducted under low NH_3_ conditions (<4 parts per trillion by volume, pptv) at the CERN Cosmics Leaving OUtdoor Droplets (CLOUD) chamber, we find that RH critically regulates the participation of MSA in the initial growth of newly formed particles. Between +10 °C and −10 °C, MSA drives rapid particle growth at high RH (>50%), whereas its contribution at low RH (<16%) is negligible. When comparing with aerosol process models such as the Model for Acid–Base Chemistry in Nanoparticle Growth (MABNAG), we find that the model fails to match our laboratory results. In particular, our measurements show that MSA drives rapid particle growth at substantially warmer temperatures and lower relative humidities than predicted by the Extended Aerosol Inorganic Model (E-AIM). This highlights the importance of further experiments to fully quantify the effect of RH on MSA particle growth and evaporation, and incorporating these measurements in aerosol models. This will be essential for accurately representing the important role of MSA in marine aerosols in global models, particularly in cold, low-NH_3_ environments like polar regions and the free troposphere.

## Abstract 2

High relative humidity substantially enhances MSA-driven growth of sub-10 nm particles, even under low-ammonia conditions.

## Graphical abstract

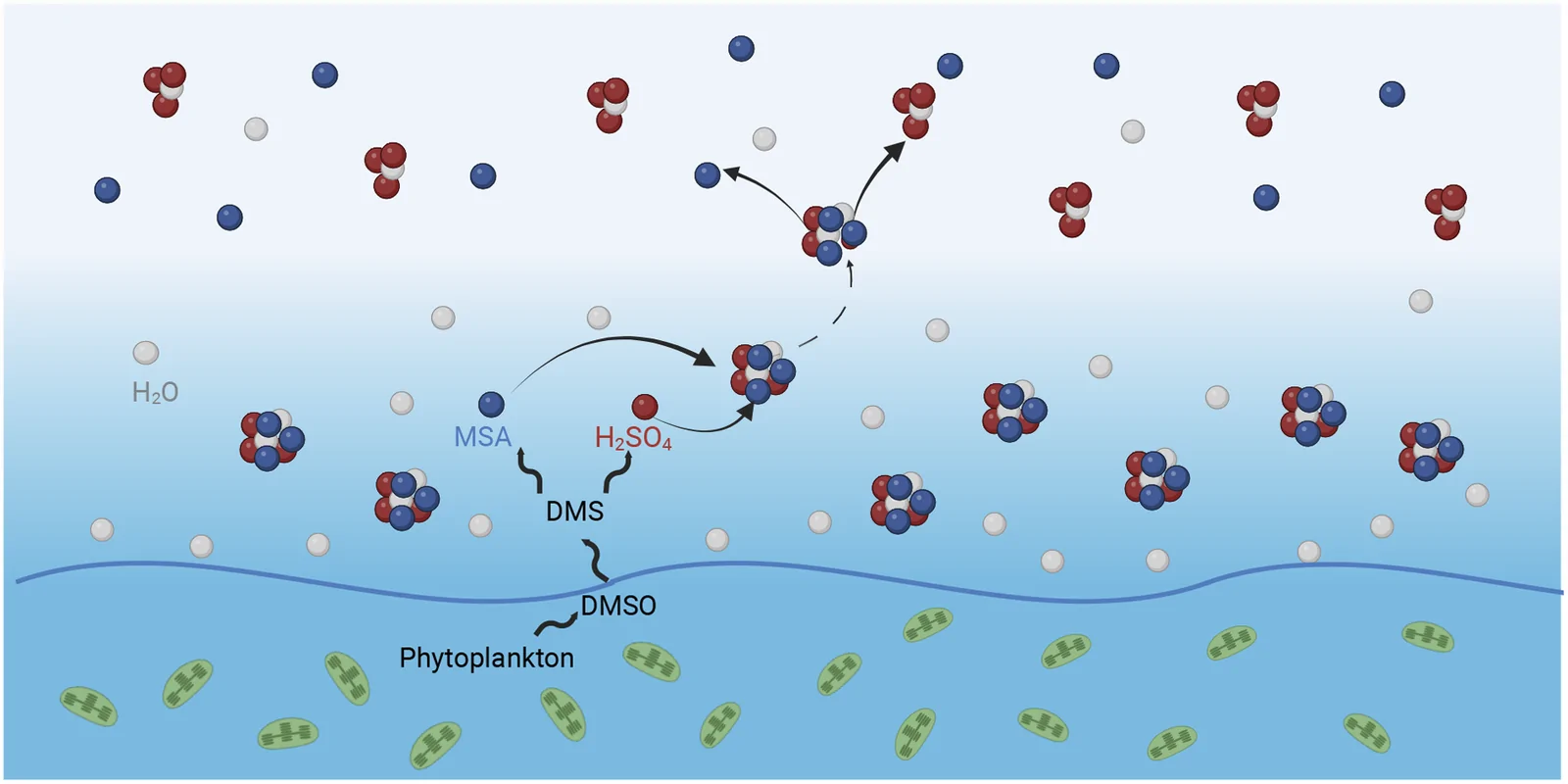

**Environmental significance**

Methanesulfonic acid (MSA) contributes to atmospheric new particle formation and growth, particularly in marine and polar environments, where it can have a significant impact on the availability of cloud condensation nuclei. The role of relative humidity (RH) in MSA condensation has remained poorly constrained and is not correctly represented in current models. Our laboratory experiments show that RH critically regulates MSA condensation in conditions representing pristine polar and free-tropospheric environments, providing essential constraints for improving the representation of aerosol growth in climate models.

## 1 Introduction

The ocean absorbs approximately 90% of the excess heat trapped by greenhouse gases,^1^ underscoring its major role in Earth's energy balance. Marine clouds influence this balance by modifying the planetary albedo over the ocean.^2^ In the remote marine atmosphere, marine cloud formation is primarily limited by the availability of cloud condensation nuclei (CCN), part of which originate from new particle formation.^3–5^ A major natural source of marine CCN is the organic sulfur compound, dimethylsulfide (DMS; CH_3_SCH_3_).^6,7^

DMS is emitted into the atmosphere through the degradation of phytoplankton-produced dimethylsulfoniopropionate.^6,8,9^ Laboratory and field studies show that two major terminal products of DMS oxidation – sulfuric acid (H_2_SO_4_) and methanesulfonic acid (MSA; CH_3_SO_3_H)^6,10–13^ – contribute to particle formation and early growth in the marine and polar atmosphere.^14–19^ In contrast to the well-studied particle formation pathways involving H_2_SO_4_, the contribution of MSA to marine CCN formation remains poorly understood. This is a critical knowledge gap since MSA is consistently observed at high concentrations between 10^5^ to 10^7^ cm^−3^ in the marine atmosphere.^19,20^ These concentrations are similar to those of other important aerosol precursors, including H_2_SO_4_ and iodine oxoacids, highlighting the need for further study of MSA's atmospheric role.^21–23^ Previous studies focused primarily on the contribution of MSA to the growth of pre-existing particles, rather than to the formation process or growth of particles below 20 nm.^14,24^ However, recent field observations in the Arctic and Southern Ocean suggested that MSA might contribute to particle initial growth (<20 nm),^19,20^ although a molecular understanding is lacking. A recent laboratory study found that, below +10 °C, MSA contributes to the growth of particles above 1.8 nm diameter and, below −10 °C, MSA drives particle nucleation in the presence of NH_3_ and H_2_SO_4_.^23^ These findings highlight the potential importance of MSA in new particle formation and growth, particularly in pristine polar regions and the marine atmosphere.

Beyond vapour concentrations, environmental factors such as temperature and RH also significantly influence new particle formation and growth. It is well-established that lower temperatures promote new particle formation from MSA, H_2_SO_4_, and oxidised organic vapours.^23,25,26^ Additionally, reduced volatility of oxidised organic vapours at low temperatures also enhances particle growth rates.^27^ In contrast to the temperature effect, higher relative humidity generally promotes particle formation, but the effect is poorly quantified. It was found that raising the RH from 20% to 90% in the H_2_SO_4_–NH_3_ system enhanced aerosol nucleation rates by several orders of magnitude.^28^ Minimal evaporation was observed from H_2_SO_4_ particles over a wide RH range, *i.e.*, the particle growth by H_2_SO_4_ is limited by the kinetic condensation rate of H_2_SO_4_.^29,30^

However, the dependence of particle growth on RH is species-dependent, and the relatively weak sensitivity of H_2_SO_4_ growth to RH cannot be directly extended to MSA. Quite the contrary, along with ground-based ambient observations,^31^ aircraft measurements provide evidence of MSA evaporation from particles under dry conditions. The aircraft measurement during the Pacific Exploratory Mission Tropics (PEM-Tropics-A) observed high MSA concentration (up to approximately 10^8^ cm^−3^) in the free troposphere, where NH_3_ concentrations were low and the temperature was −3 °C, with the highest MSA observed at low RH (<20%).^32^ Furthermore, the same observations showed that the concentration of gaseous MSA increases significantly from 6 × 10^5^ to 2.2 × 10^7^ cm^−3^ at low relative humidity (approximately 40%), while particle-phase MSA decreases.^33^ These field observations suggest that MSA's contribution to particle growth is influenced by ambient RH. While H_2_SO_4_ is essentially completely miscible with water, MSA also exhibits high solubility,^34^ with Henry's law constants reported as 6.5 × 10^11^ mol per kg per atm.^35^ This indicate that MSA, like H_2_SO_4_, has a strong thermodynamic tendency to partition into the aqueous phase. However, subtle differences in hydration energetics and cluster stability may explain why RH exerts a stronger influence on MSA in the particle phase than H_2_SO_4_. Quantum chemical calculations show that MSA–H_2_O clusters exhibit weaker hydration free energies, earlier proton transfer induced destabilization, and faster water evaporation than H_2_SO_4_–H_2_O clusters, which maintain stronger binding and only form ion pairs at larger hydration numbers.^36^ This influences cluster formation pathways and potentially affects the particle initial growth at different relative humidity.

In addition to field observations, several flow tube experiments have investigated the role of MSA in new particle formation and growth and its RH effect. Kreidenweis *et al.*^37^ examined MSA–H_2_O nucleation and reported that the measured number concentration of particles >30 nm increased by a factor of 10 000 as RH rose from 10% to 50%. However, the experimental conditions – particularly the MSA concentration and NH_3_ levels – were not documented. Subsequent flow tube studies have shown that RH enhanced particle nucleation rates in MSA nucleation with alkaline molecules by a factor of two to five, depending on the specific base involved such as NH_3_, trimethylamine, and dimethylamine.^38,39^ In these experiments, the concentrations of MSA (3 × 10^10^ to 5 × 10^10^ cm^−3^), NH_3_ (18 ppbv), trimethylamine (2.5 ppbv), dimethylamine (2.5 ppbv) were far higher than atmospherically relevant levels, making it challenging to infer implications for real atmospheric conditions. In contrast, experiments on acid uptake by acidic particles, conducted in a laminar flow tube at atmospheric pressure, found that both MSA and H_2_SO_4_ have similar mass accommodation coefficients, close to unity, over a wide RH range (6% to 97%) at room temperature.^40^ This suggests that the initial kinetic step of uptake is efficient and only weakly dependent on RH. However, the mass accommodation coefficient primarily describes the kinetics of gas-to-particle transfer and does not capture the thermodynamic factors governing equilibrium partitioning or nucleation. Therefore, while the mass accommodation may be comparable for MSA and H_2_SO_4_, this does not preclude the possibility that RH strongly influences nucleation rates and particle growth, as reported in earlier flow tube studies and ambient measurements.^32,37–39^

In addition to laboratory experiments and ambient observations, theoretical studies have also examined the enhancing effect of H_2_O on MSA particle nucleation. For example, a theoretical study using quantum chemical calculations and kinetics simulations found that hydration enhances the formation of MSA–NH_3_ clusters by a factor of 10^5^ compared to dry conditions.^41^ Moreover, the formation of MSA–NH_3_–H_2_O clusters becomes increasingly important at RH above 40%, where the evaporation rate of the MSA–NH_3_ dimer is significantly reduced.^41^ Other theoretical studies,^42,43^ focusing on the H_2_SO_4_–H_2_O–MSA system (in the absence of bases), show that MSA–H_2_SO_4_–(H_2_O)_*n*_ clusters are more stable than MSA–H_2_SO_4_ alone. This indicates that H_2_O promotes nucleation and may enhance early particle growth even without NH_3_, consistent with aircraft measurements in the free troposphere where NH_3_ concentrations were low.^44^

A combination of ambient observations, laboratory experiments, and theoretical studies clearly demonstrates the significant role of water vapour in influencing MSA-driven particle nucleation and growth. However, a quantitative understanding of these processes under atmospherically relevant conditions remains limited. Earlier laboratory studies often struggled to reduce NH_3_ concentrations to ambient levels due to experimental contamination. The presence of NH_3_ complicates the interpretation of RH effects, as it stabilises particulate acidic species and reduces their evaporation, particularly, in the case of H_2_SO_4_.^29^ Consequently, the influence of RH on MSA's contribution to early particle growth under low- or no-NH_3_ conditions remains poorly constrained. This knowledge gap is particularly important for understanding particle formation and initial growth in both polar regions and the free troposphere, where NH_3_ is scarce and MSA levels can be elevated due to low temperatures that favour its production from DMS oxidation.^13^

Here we report experiments performed at the Cosmics Leaving Outdoor Droplets (CLOUD) chamber at CERN^25^ to investigate the influence of RH on the initial growth of particles from MSA under polar and free tropospheric conditions. The experiments were performed between −30 °C and +10 °C and at RH values between 16% and 100%. Most experiments were conducted without NH_3_ injection to maintain contaminant levels below 4 pptv, to mimic pristine marine environments where NH_3_ concentrations are typically low.^44^ The experimental results are used to assess the accuracy of MSA volatility predictions by the widely-used Extended Aerosol Inorganics Model (E-AIM), which accounts for both temperature and RH. In addition, we apply the thermodynamic particle growth model, Model for Acid–Base Chemistry in Nanoparticle Growth (MABNAG) to simulate growth under the same conditions and compare with the experimental measurements.

## 2 Material and methods

### 2.1 CLOUD experiments

The data used in this study were collected during the CLOUD16 and CLOUD17 campaigns, from September to November 2023 and 2024, respectively, at the CLOUD experiment at CERN, Switzerland. Additionally, two experimental runs from the CLOUD14 and CLOUD15 campaigns, as reported by Baalbaki *et al.*,^23^ are included.

The CLOUD chamber at CERN is a 26.1 m^3^, stainless-steel, electropolished chamber to study new particle formation under well-controlled conditions, representing boundary layer, tropospheric, and lower-stratospheric conditions.^25,45^ A thermal housing around the chamber maintains a stable temperature over a wide range (208 to 373 K with high precision (±0.1 K)).^46^ The chamber runs continuously, supplied with ultra-pure synthetic air generated by mixing cryogenic liquid oxygen and liquid nitrogen in proportions of 20% O_2_ and 80% N_2_, which are steadily injected into the chamber. DMS (1000 ppmv in N_2_) was continuously injected into the chamber, regulated by mass flow controllers (MFCs) and multiple dilution steps. Ozone (O_3_) was introduced into the chamber by flowing pure O_2_ through an ozone generator. Inside the chamber, O_3_ was photolysed using a combination of four 200 W Hamamatsu mercury–xenon (Hg–Xe) ultraviolet lamps emitting wavelengths between 250 and 450 nm, four low-pressure mercury (Hg) lamps operating primarily at 254 nm, along with a xenon fluoride (XeF) excimer laser (UVX) operating at 248 nm, to generate hydroxyl (OH) radicals. Additionally, to minimise cross-contamination, especially concerning NH_3_ here, the chamber undergoes a cleaning procedure between experimental programmes. This involves rinsing the chamber walls thoroughly with ultra-pure water, followed by heating the chamber to 373 K for over 24 hours.

Different ionisation settings can be used to study the ion effect on new particle formation at the CLOUD chamber. In the so-called neutral mode, a powerful electric field (±30 kV) is used, rapidly eliminating all small ions from the chamber to establish an ion-free environment. In ground-level galactic cosmic ray (GCR) experiments, the electric field is off so natural ions are generated in the chamber by galactic cosmic rays. The ion production rate under GCR conditions inside the chamber has previously been determined to range from 1.7 to 4.1 ion pairs cm^−3^ s^−1^.^26,47^ Uniform mixing of particles and vapours inside the chamber is accomplished using two magnetically coupled stainless-steel fans located at the top and bottom of the chamber. This configuration ensures rapid and efficient mixing within around two minutes.

To investigate the role of MSA in particle initial growth under a range of RH conditions, we conducted experiments at temperatures of +10 °C, −10 °C and −30 °C with RH from 16% to 100%. Before each experiment, precursors (DMS and O_3_) were injected into the chamber; their concentrations were stabilised under dark conditions with fans running at maximum speed (100%) to ensure complete mixing. Once stable precursor concentrations and particle-free conditions were achieved, the fan speed was reduced to 12%, and the chamber lights were switched on to initiate experiments. Switching on the lights initiated photochemical production of OH radicals, leading to oxidation of DMS and subsequent formation of MSA and H_2_SO_4_. The light intensity was varied to control the concentrations of MSA and H_2_SO_4_. Following particle generation in the chamber, the experiment was continued until a stable nucleation rate and the desired particle growth, with particles growing large enough, were achieved under steady state conditions.

### 2.2 Instrumentation

The relative humidity is calculated from a chilled-mirror dew-point Hygrometer (DewMaster; Edgetech Instruments). The DewMaster measures the dew point (above 0 °C) or frost point (below 0 °C) in the chamber. Relative humidity is then calculated as the ratio of the saturation vapour pressure at the dew point or frost point to the saturation vapour pressure at the actual chamber temperature, expressed as a percentage by multiplying by 100.

The concentrations of MSA and H_2_SO_4_ in the chamber were measured using the nitrate chemical-ionisation mass spectrometer (NO_3_^−^-CIMS; Tofwerk AG, Thun, Switzerland) coupled with a corona nitrate ion source.^48^ The instrument was calibrated through H_2_SO_4_ calibration experiments, as described in detail in previous studies.^48–50^ The calibration coefficients applied for the CLOUD16 and CLOUD17 campaigns are 1.25 × 10^10^ and 1.06 × 10^10^ cm^−3^ per normalised signal (cps per cps; the ratio of the analyte ion to reagent ion signals; cps denotes counts per second), with a systematic error ranging from 66% to 150%. The same calibration factor was applied to MSA, based on its similar collision-limited charging efficiency to that of H_2_SO_4_.

To analyse the chemical composition of particles, a Filter Inlet for Gases and AEROsols (FIGAERO) coupled to a chemical-ionisation mass spectrometer was used.^51^ The particles were collected from the chamber onto a 5 µm-pore polytetrafluoroethylene (PTFE) filter (MilliporeSigma), and then subjected to a controlled heating process to thermally desorb volatile molecules into the gas phase. The mass loading on the filter depends on the particle size distribution in the chamber, the flow rate (7 l min^−1^), and the collection time (1 hour). FIGAERO-CIMS was operated using Br^−^ chemical ionisation^52^ in a reduced-pressure ion molecule reactor in several of the experiments presented: +10 °C under both dry and humid conditions, −10 °C with 74% RH, and −30 °C with 59% RH. It was operated using I^−^ chemical ionisation during CLOUD17, for experiments conducted at −10 °C, 16% RH, and −30 °C, 100% RH. Since MSA and H_2_SO_4_ were not independently calibrated for FIGAERO-CIMS, it remains uncertain whether the instrument's sensitivity to these two compounds is equivalent. Therefore, comparisons of MSA and H_2_SO_4_ in the particle phase should be interpreted qualitatively rather than quantitatively.

Several instruments were employed to measure particle and ion number size distribution from approximately 1 nm to 1000 nm. The particle number size distribution between 1 and 3 nm was measured with a scanning Particle Size Magnifier (PSM; Airmodus) coupled with a butanol Condensation Particle Counter (CPC).^53^ The PSM used diethylene glycol (DEG) to grow small particles to a size that can be detected by a CPC. The Neutral cluster and Air Ion Spectrometer (NAIS; Airel)^54^ was used to measure the number size distribution of ions ranging from 0.8 to 42 nm in both polarities, as well as the total particle size distribution from 2 to 42 nm. Like other electrical mobility spectrometers, the NAIS classifies ions based on electrical mobility. A nano-Scanning Mobility Particle Sizer (TSI3938) (nano-SMPS)^55^ coupled with a butanol CPC (TSI3776) was used to measure the particle number size range between 6 and 65 nm, while the particles larger than 65 nm, up to 1000 nm, were measured with a commercially available long-mobility Scanning Mobility Particle Sizer (long-SMPS) (TSI 3082) coupled to a butanol CPC (TSI 3775).^56^

The nucleation rate, *J*_1.7_, is calculated based on the PSM measurements by taking the derivative of the total particle concentration above 1.7 nm 
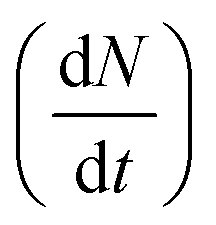
 and accounting for the CLOUD chamber specific losses of particles due to dilution *S*_dil_, wall loss *S*_wall_ and coagulation *S*_coag_. The nucleation rate, *J*_1.7_ is thus defined as1
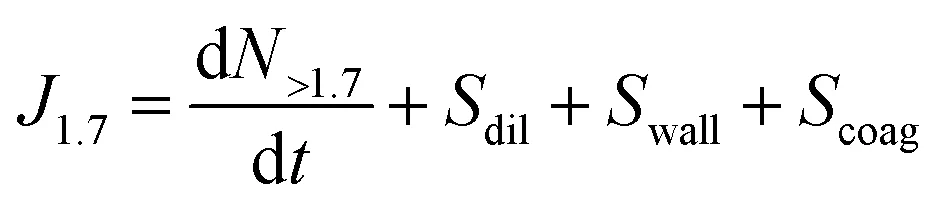


The coagulation sink is derived from the combined particle number size distribution measured by the NAIS and SMPS instruments as described in our previous study.^57^

The particle number size distribution from NAIS is used to calculate growth rates (GR) between 3.2 and 8.0 nm, using the 50% appearance time method,^58^ which was theoretically derived in He *et al.*^47^ The appearance time method may overestimate the growth rate when coagulation contributes significantly to particle growth relative to condensation.^47,59^ However, this effect is expected to be small in these experiments.

### 2.3 NH_3_ measurement and estimation

Accurate NH_3_ measurements are critical to mimic concentrations found in the free troposphere and marine atmosphere. The NH_3_ concentration during the experiment in CLOUD14 campaign was 246 pptv according to measurements with the H_3_O^+^-PTR3-CIMS, as reported by Baalbaki *et al.*^23^ NH_3_ concentration was estimated to be between 4 and 110 pptv for the experiment presented here in CLOUD15,^23^ based on the amount of NH_3_ injected into the chamber and the calculated wall loss rate. During CLOUD16, no NH_3_ was injected into the CLOUD chamber after a cleaning procedure with ultra-pure water and a heating cycle. Consequently, the NH_3_ concentration is expected to reflect the chamber's background level, which is typically below 4 pptv, based on a previous study.^60^ For the prior experiments in CLOUD17, NH_3_ injections had been performed, and a full cleaning cycle was not carried out. However, a Tunable Infrared Laser Direct Absorption Spectroscopy (TILDAS, Aerodyne Research Inc.) was used to confirm that the NH_3_ concentrations in the chamber remained below detection limit (approximately 20 pptv) during the experiments presented here. It should be noted that TILDAS was used solely to ensure that NH_3_ levels did not unintentionally exceed its detection limit; it was not intended to provide precise NH_3_ concentration measurements across all experiments. The actual NH_3_ concentrations were likely much lower, as indicated by the cluster measurements with an Atmospheric Pressure Interface Time-of-Flight Mass Spectrometer (APi-TOF, Aerodyne Research Inc.), which provides the most sensitive qualitative indicator of NH_3_ presence in the chamber. Even trace amounts of NH_3_, *e.g.*, a few pptv, would result in abundant H_2_SO_4_–NH_3_ cluster formation.^61^ Therefore, the absence of such clusters in our experiments strongly suggests that the NH_3_ concentrations during our experiments were likely below 4 pptv in CLOUD17.

### 2.4 Model description

Model for Acid–Base chemistry in Nanoparticle Growth (MABNAG) simulates the growth and composition of a single particle upon condensation of vapours and accounts for acid dissociation and base protonation in the particle phase.^62^ The particle phase acid–base chemistry and composition-dependent equilibrium vapour pressures are calculated in MABNAG with the E-AIM.^63–66^ The condensing vapours considered in the model simulations are H_2_SO_4_, MSA and H_2_O. In MABNAG, the condensation of acids is calculated based on their condensation mass fluxes, while the condensation of water is calculated by assuming continuous gas–particle equilibrium. The particles are assumed to be in the liquid phase. In MABNAG, MSA is treated either as MSA itself or as H_2_SO_4_ in this study to evaluate whether these representations can reproduce the observed growth in experiments. MSA's dissociation constant is assumed to be 79.4 mol kg^−1^, with saturation vapour pressure at 298.15 K as 5.63 × 10^−7^ atm, and enthalpy of vaporization of 50 kJ mol^−1^.^67^ The neutral form of the MSA in particle phase is treated as an ideal mixture in E-AIM coupled in the MABNAG, while E-AIM uses the interaction parameters of HSO_4_^−^ for singly charged MSA anions. The particle density and surface tension are assumed to be 1500 kg m^−3^ and 0.03 N m^−1^, respectively.^62^ The gas phase diffusion coefficient was 10^−5^ m^2^ s^−1^. The simulations are initialised with particles consisting of 6 molecules of sulfuric acid (particle diameter approx. 1.01 nm). Gas concentrations of H_2_SO_4_ and MSA, temperature and RH are set constant through a simulation according to the corresponding experimental conditions. The growth rates from simulated data are calculated based on the dry size of the particle for the size range 3.2–8 nm to match the experimental data.

The volatility of MSA is affected by the temperature, RH and availability of bases.^68–70^ E-AIM is a thermodynamic model that has previously been applied to estimate the volatility as a function of temperature, RH, and the presence of gas-phase bases.^63,64,66^ In this study, we compare our results with the transitional parameterisation proposed by Hodshire *et al.*,^67^ which characterises whether MSA behaves as “ELVOC-like species”, condensing irreversibly to aerosol of all sizes, or as “SVOC-like species”, where the net condensation of MSA is proportional to aerosol mass distribution. In that study, E-AIM Model II is used to characterise the thermodynamic equilibrium of the H^+^–nssSO_4_^2−^–NO_3_^−^–H_2_O system, which is used here as a representative secondary aerosol composition. The transition line between these two ideal cases is given by

2 *T*_trans_(RH) = 2.52 × 10^2^ − 6.19 × 10^−1^ × RH + 3.49 × 10^−2^ × RH^2^ − 5.6 × 10^−4^ × RH^3^ + 3.32 × 10^−6^ × RH^4^ − 273.15

where RH is the relative humidity, *T* is the temperature (°C), and *T*_trans_ is the transition temperature. When *T* > *T*_trans_, MSA is considered to contribute primarily to the particle mass instead of promoting particle formation and initial growth. Conversely, when *T* < *T*_trans_, MSA is considered to contribute directly to both particle nucleation and initial growth.

## 3 Results and discussion

### 3.1 Observed particle growth under varying RH

**Fig. 1:**
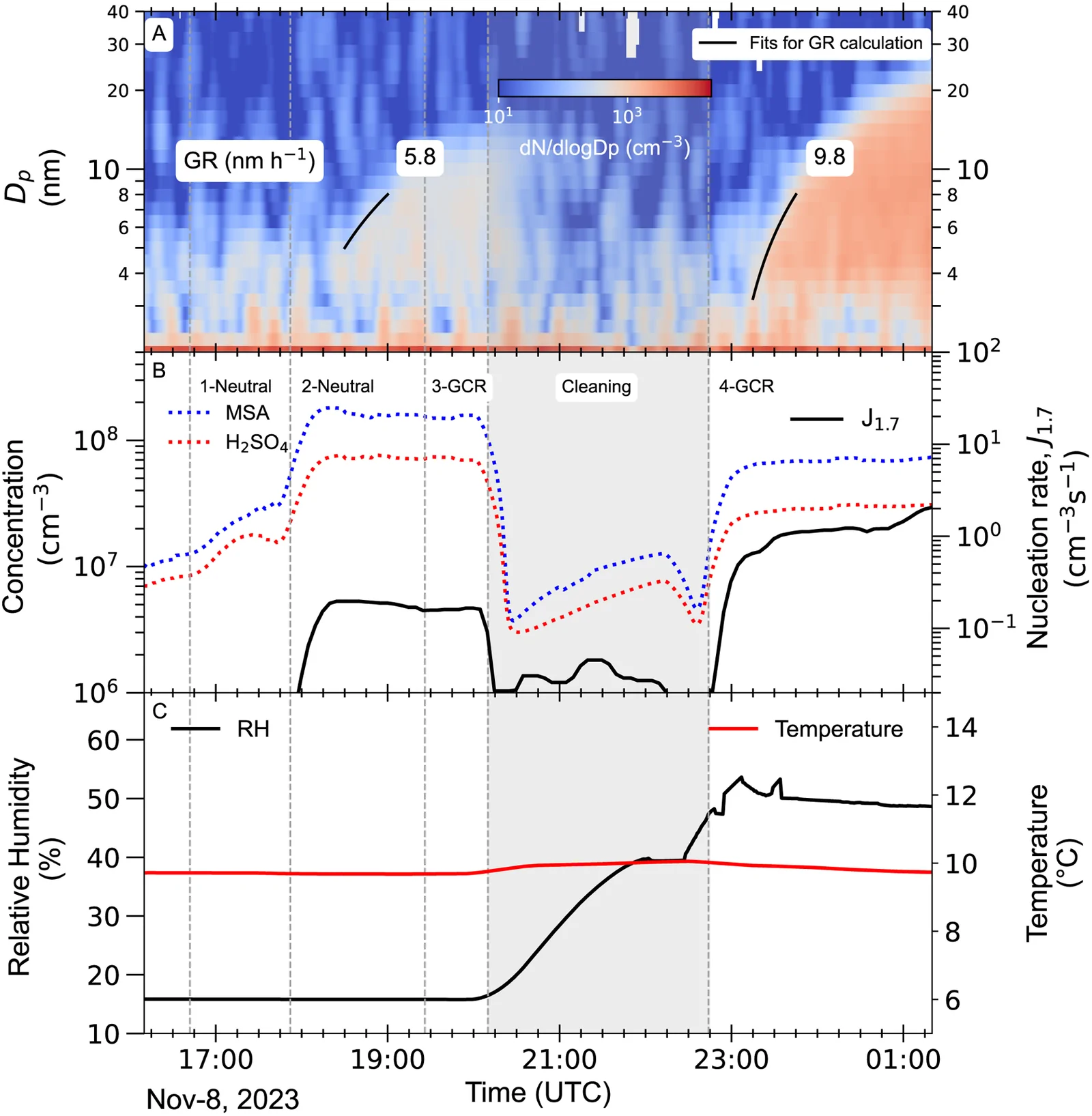
Particle formation experiment from MSA and H_2_SO_4_ at low and high RH, at +10 °C. (A) Particle number size distribution and growth rate measured between 3.2 and 8 nm for total particles from the NAIS. The black lines in panel A show the 50% appearance time of particles between 3.2 and 8 nm. (B) Evolution of vapour concentration and particle nucleation rate at 1.7 nm, *J*_1.7_. (C) Temperature and relative humidity. The vertical grey dashed lines indicate the start of a new stage, during which the experimental conditions are adjusted. Stage 1 and 2 are, under neutral (ion free) conditions, whereas stage 3 and 4 are under galactic cosmic ray (GCR; natural ion concentrations) conditions. The grey-shaded area represents a cleaning stage, where the fan speed is increased from 12% to 100%, and the lights are turned off.

Fig. 1 presents an example of two sequential particle formation and growth experiments involving H_2_SO_4_ and MSA, conducted at +10 °C under two different RH levels, 16% and 50% RH. The experiment begins at 16% RH with initial concentrations of 10^7^ per cm^3^ MSA and 6 × 10^6^ per cm^3^ H_2_SO_4_, both produced *via* OH-initiated oxidation of DMS (stage 1-Neutral in Fig. 1). No particle formation is observed at this stage, as the acid concentrations are too low to initiate nucleation in the absence of bases. During these experiments, the NH_3_ concentration is assumed to be below 4 pptv, as abundant H_2_SO_4_–NH_3_ ionic clusters are absent from the ion spectra measured by the APi-TOF.^61^ The absence of NH_3_-containing clusters indicates that NH_3_ levels remained low throughout the measurements.

Increasing the light intensity enhances the OH production rate, which in turn raises MSA and H_2_SO_4_ concentrations to 1.58 × 10^8^ cm^−3^ and 7.39 × 10^7^ cm^−3^, respectively, triggering new particle formation (stage 2-Neutral in Fig. 1). The particle growth rate in the 3.2–8 nm size range is 5.8 nm h^−1^. This growth rate can be explained by H_2_SO_4_ condensation alone, as the expected growth rate from H_2_SO_4_ in this stage is 5.5 nm h^−1^.^30,71^ This suggests that the contribution of MSA to particle growth is negligible or very small at this RH. Between stage 2-Neutral and stage 3-GCR (around 19:30 in Fig. 1), the ionisation condition in the chamber is changed from neutral to GCR, allowing ions to form in the chamber. However, the particle nucleation rate does not exhibit any significant change under these conditions. At +10 °C, H_2_SO_4_–H_2_O(–NH_3_) nucleation is generally dominated by ion-induced mechanisms.^28^ The absence of a clear ion enhancement in our experiments may suggest the presence of trace amounts of amines in the chamber, as particle nucleation involving H_2_SO_4_ and amines is known to proceed primarily through the neutral channel.^72^ Amines effectively stabilise nascent acid clusters, thereby suppressing ion-induced contributions to the nucleation rate.^72^ Although amines were not detected by the APi-TOF instrument (detection limit well below 10^7^ cm^−3^) – contamination-level amine concentrations cannot be entirely excluded. Nevertheless, the estimated H_2_SO_4_ : amine ratio under these conditions exceeds 20 : 1, indicating that any potential amine presence is extremely low relative to acid levels. Consequently, such low amine concentrations are unlikely to affect our conclusions regarding particle early growth. In general, ions are expected to have a minor effect on the growth rates at sizes beyond 3 nm.^73^

After the particle nucleation rate reaches steady state in stage 3-GCR, the experiment is terminated by turning off the lights and increasing fan speed to 100% to remove existing particles. During this cleaning stage (grey-shaded area in Fig. 1), the RH was adjusted from 16% to 50% and O_3_ concentration was decreased to prepare for the next stage. Once all particles are removed, a new experiment is conducted at 50% RH under GCR conditions. With lower light intensity and lower O_3_ concentration, the OH production rate is lower compared to previous stages, resulting in lower acid concentrations of 6.70 × 10^7^ per cm^3^ MSA and 2.77 × 10^7^ per cm^3^ H_2_SO_4_ (stage 4-GCR in Fig. 1). Therefore, the total acid concentration in stage 4-GCR is lowered by a factor of 2.5 compared to stage 3-GCR. Surprisingly, the observed growth rate increases to 9.8 nm h^−1^ despite the lower vapour concentration. Given the measured H_2_SO_4_ concentration of 2.77 × 10^7^ cm^−3^, the expected particle growth rate attributable to H_2_SO_4_ alone is approximately 3.1 nm h^−1^,^30^ which is significantly lower than the observed growth rate. This discrepancy indicates that additional condensable vapours must be contributing to the growth, with MSA being the most likely candidate here. Assuming an MSA accommodation coefficient of unity at 50% RH, and using the parameterisation for particle growth rate from 3.2 to 8 nm by Stolzenburg *et al.*,^30^ the condensation of MSA at a concentration of 6.70 × 10^7^ cm^−3^ can quantitatively account for the remaining growth (6.7 nm h^−1^). This supports the participation of MSA in particle growth at 50% RH.

In addition to the experiments conducted at +10 °C, we performed experiments at −10 °C and −30 °C to simulate atmospheric conditions representative of polar regions and the free troposphere (Table 1). The results reveal a consistent trend: at −10 °C, the contribution of MSA to particle initial growth seems to be also dependent on relative humidity. Specifically, at 16% RH, the observed growth rate is only 1.8 nm h^−1^, which can be largely attributed to H_2_SO_4_ alone, indicating a negligible role for MSA under relatively dry conditions. However, at 74% RH, the observed growth rate is 3.1 nm h^−1^ – substantially exceeding the contribution expected from H_2_SO_4_ alone. This implies that MSA plays an important role in particle growth under humid conditions. Due to experimental limitations, we were unable to perform experiments at low RH at −30 °C. The difference in growth rates between 59% RH (1.8 nm h^−1^) and 100% RH (4.0 nm h^−1^) can be explained by different acid concentrations, so most likely MSA participated in particle growth at both RH conditions (Table 1) as both values are much higher than what H_2_SO_4_ condensation alone can explain.

**Table 1:** Comparison between the measured and simulated growth rates from 3.2 to 8 nm under different temperature and humidity (RH) conditions

Campaign	Temperature (°C)	RH (%)	MSA concentration (cm^−3^)	H_2_SO_4_ concentration (cm^−3^)	NH_3_ Concentration (ppt)	GR CLOUD (nm h^−1^)	GR MABNAG without MSA (nm h^−1^)	GR MABNAG with MSA (nm h^−1^)	GR MABNAG MSA as sulfuric acid (nm h^−1^)	GR MABNAG without MSA/GR CLOUD ratio (%)
CLOUD15 (ref. 23)	−30	59	5.22 × 10^6^	4.45 × 10^5^	4–110	1.8	0.1	0.1	0.6	5.6
CLOUD17	−30	100	1.15 × 10^7^	4.61 × 10^5^	<4	4.0	0.1	0.1	2.3	2.5
CLOUD17	−10	16	5.68 × 10^7^	1.64 × 10^7^	<4	1.8	1.3	1.3	5.8	2.2
CLOUD14 (ref. 23)	−10	74	2.64 × 10^7^	5.92 × 10^6^	246	3.1	0.7	0.7	3.6	22.6
CLOUD16	+10	16	1.58 × 10^8^	7.39 × 10^7^	<4	5.8	5.9	8.9	18.6	100.2
CLOUD16	+10	50	6.70 × 10^7^	2.77 × 10^7^	<4	9.8	2.7	2.7	9.2	27.6

**Fig. 2:**
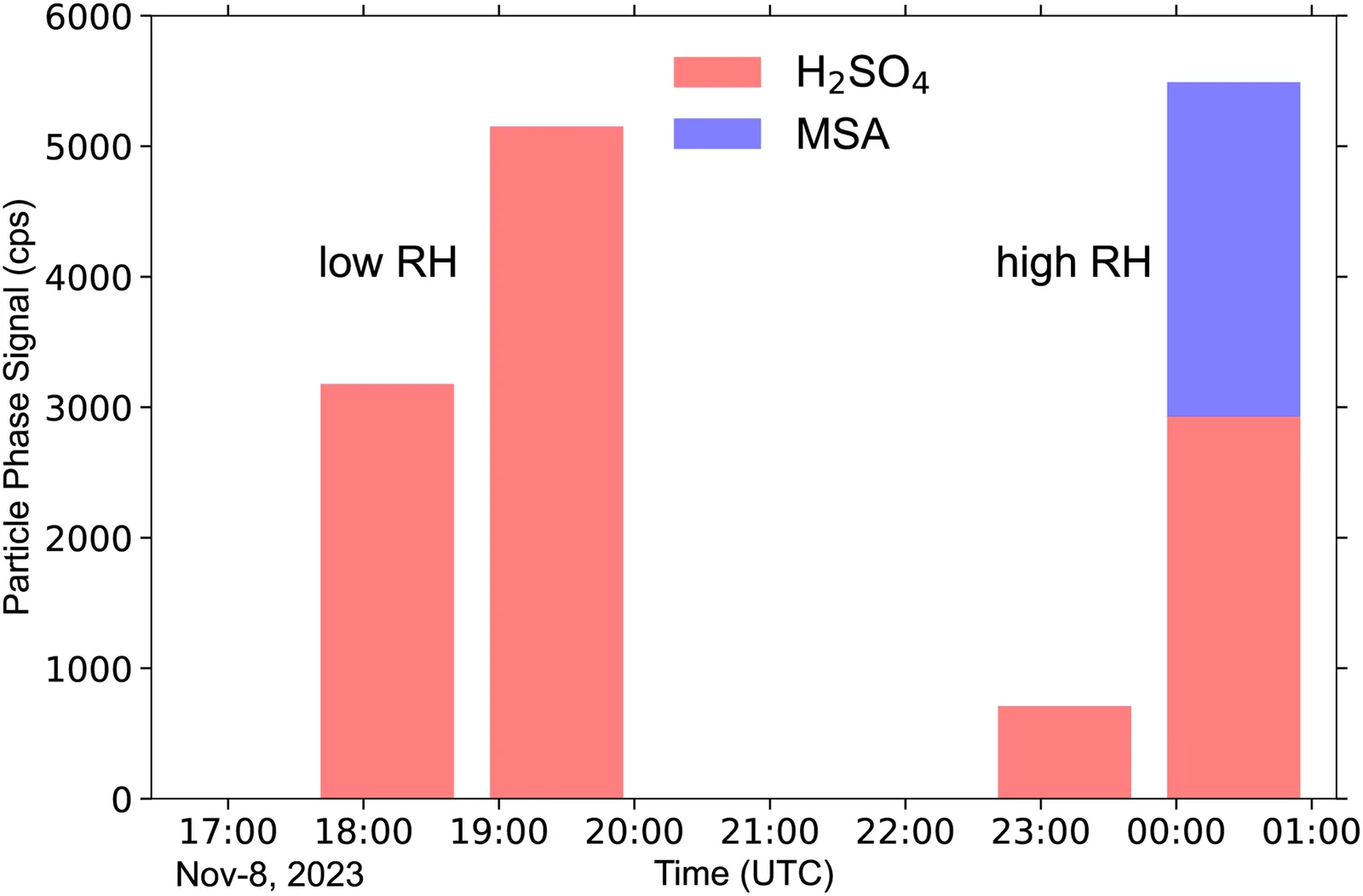
Particle-phase H_2_SO_4_ and MSA signals during the experiment shown in Fig. 1. Each sample from the Br^−^-FIGAERO-CIMS was collected over a one-hour period and then thermally desorbed for approximately 15 minutes to obtain the signals. The bin width in the figure represents the filter collection period and the bin height represents the integrated ion signal over the desorption period. The left section corresponds to the low relative humidity (16%) stage during which the mean particle size is 6.5 nm, while the right section corresponds to the high relative humidity (50%) stage, with a mean particle size of 5.6 nm.

Direct particle-phase measurements using the FIGAERO-CIMS provide additional independent evidence for MSA's involvement in particle growth, as shown in Fig. 2. This figure presents the particle-phase signals of MSA and H_2_SO_4_ from the Br^−^-FIGAERO-CIMS during stages 3-GCR and 4-GCR of Fig. 1, which correspond to periods with sufficient particle mass loading for filter collection. Notably, during stage 3-GCR (16% RH), no particle-phase MSA signal is detected, despite the gaseous MSA concentration being much higher than that of H_2_SO_4_. This confirms that MSA does not contribute significantly to particle growth under dry conditions. In contrast, during stage 4-GCR (50% RH), MSA is clearly detected in the particle phase, indicating its involvement in particle growth at higher humidity. While this study does not attempt to quantify the absolute particle-phase mass of MSA or H_2_SO_4_, the presence of MSA in particles at 50% RH and its absence at 16% RH supports the conclusion that MSA contributes to particle growth under more humid conditions, consistent with the growth rate analysis.

### 3.2 Model simulations of particle growth in the MSA experiments

**Fig. 3:**
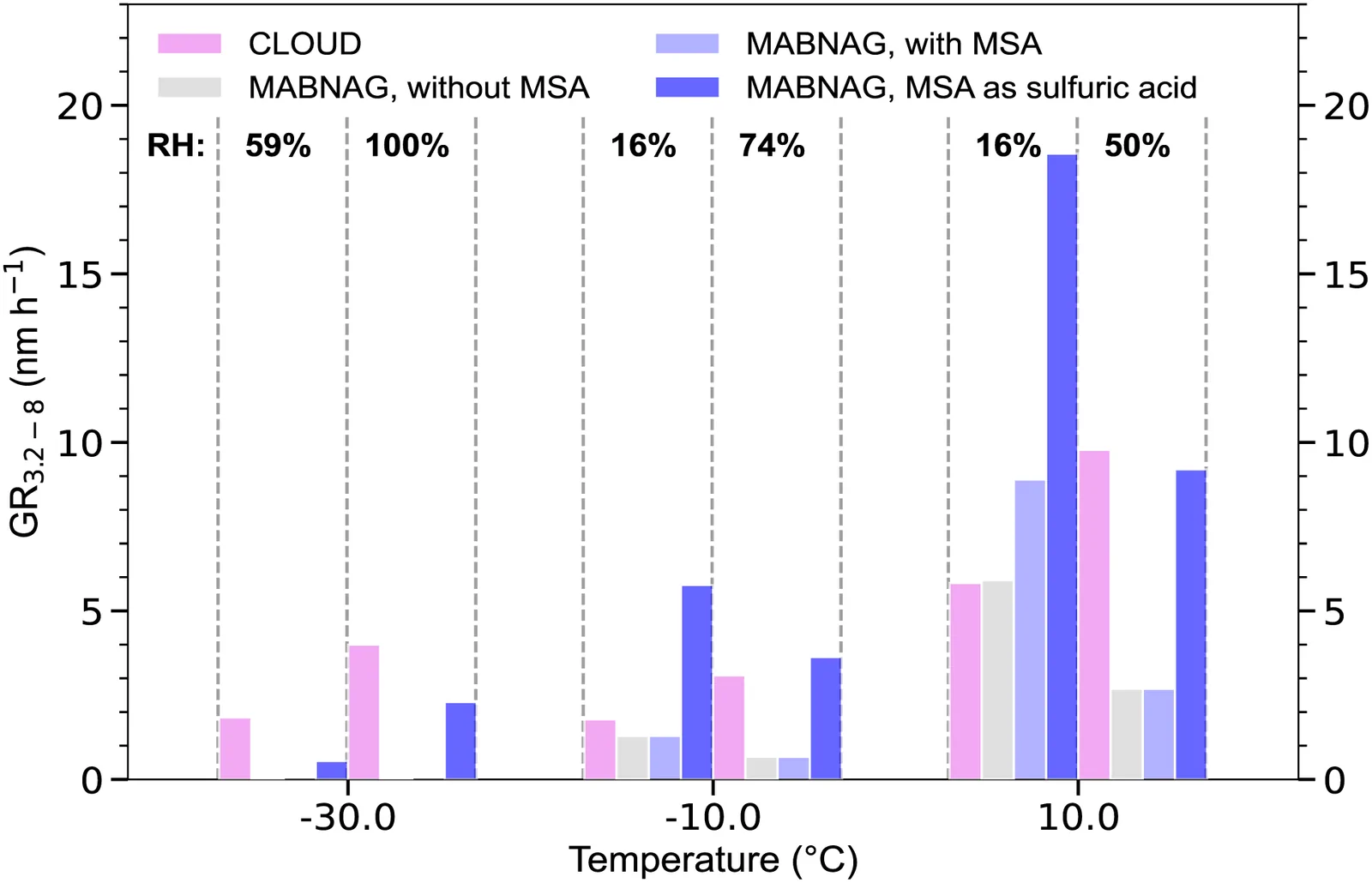
Comparison of experimental and modelled GRs, with conditions in Table 1. The concentration of precursor gases are adjusted according to the experimental conditions in the MABNAG model. The height of the bar plots represents the GR from 3.2 to 8 nm. Pink bars shows experimental measurements, while grey, light blue, and dark blue bars represent model simulations without MSA, with MSA, and with MSA treated as H_2_SO_4_, respectively. The details are shown in Table 1. The model cannot capture the growth rate partly because the influence of RH on MSA condensation is not reproduced in the model.

To further quantify the contribution of different compounds to particle growth, a particle growth model, MABNAG, is used to simulate particle growth under the same conditions as the CLOUD experiments. As shown in Table 1 and Fig. 3, the simulated growth rates from the MABNAG model without MSA (grey bars) agree well with the experimental results (pink bars) at the two low-RH experiments at −10 °C and +10 °C. These results support the conclusion that MSA has a negligible effect on particle growth under low-RH conditions. In contrast, the model significantly underestimates particle growth rates under high-RH conditions (RH > 50%) at all temperatures when MSA is excluded. This aligns with our experimental findings, which indicate that H_2_SO_4_ alone cannot fully explain the observed particle growth at high RH. While H_2_SO_4_ and highly oxidised organic molecules are typically included in models of aerosol formation and early growth, MSA is often neglected due to the long-standing assumption that it primarily contributes to the growth of larger particles.^74–76^ However, our results demonstrate that under humid conditions, MSA plays a significant role even in particle initial growth. Therefore, MSA should be explicitly included in particle growth simulations, particularly at higher relative humidity.

We explore two simplified approaches: treating MSA as itself (light blue bars in Fig. 3) or as H_2_SO_4_ (dark blue bars in Fig. 3) in the particle growth calculations. These sensitivity tests assess whether such approximations are appropriate for representing MSA's role in atmospheric particle growth. The key difference between MSA and H_2_SO_4_ is their volatility and chemical properties. H_2_SO_4_ is a strong acid (p*K*_a_ = −3) with extremely low volatility, leading to condensation onto particles at kinetic limit even at the smallest particle sizes.^30,73^ In contrast, MSA is less acidic (p*K*_a_ = −1.96) and exhibits higher volatility, resulting in relatively limited particle growth due to evaporation of MSA from the condensed phase. These sensitivity simulations are presented as the light blue (with MSA) and dark blue (MSA as H_2_SO_4_) bars in Fig. 3. When MSA is included in the model, the simulated growth rates still underestimate the experimental observations at high RH, indicating an underestimation of MSA's actual contribution. This suggests that, on a per-molecule basis, MSA-driven growth is more efficient than predicted by the simulations under high-RH conditions. Furthermore, sensitivity tests using the MABNAG model under varying surface tension show that changes in surface tension alone cannot explain the pronounced RH dependence of MSA-driven aerosol growth. Conversely, when MSA is treated as H_2_SO_4_ (the dark blue bars), the simulated growth rates are comparable to the experimental values. This improved agreement suggests that MSA resembles H_2_SO_4_ in respect to particle growth under humid conditions.

At −30 °C, all three model scenarios significantly underestimate the observed growth rates at both RH conditions (59% and 100%). MABNAG predicts a growth rate of approximately 0.1 nm h^−1^ if particle growth is only driven by H_2_SO_4_, consistent with the expected growth rates based on the Stolzenburg *et al.*^30^ This discrepancy highlights the limitations of the current thermodynamic treatment under low-temperature conditions. A possible reason may be the presence of a large number of molecular clusters at lower temperatures that might contribute to the observed growth, as suggested by earlier laboratory studies.^21,23,73^ The results presented in this study unambiguously indicate that representing MSA's growth contribution using proxy vapours such as H_2_SO_4_ or simplified parameterisations of MSA itself is inaccurate. Its quantitative role in particle growth must be assessed through dedicated, targeted investigations.

**Fig. 4:**
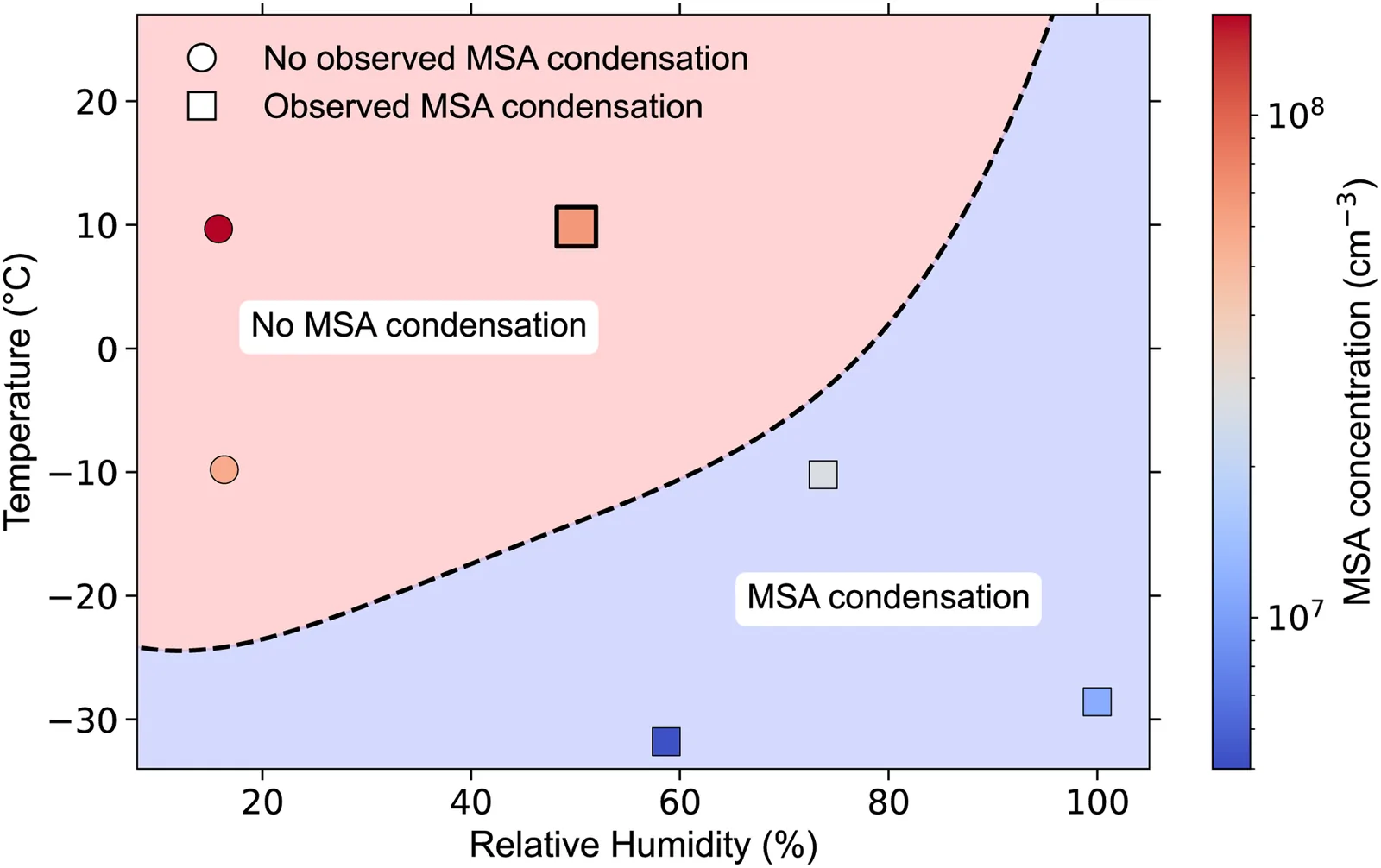
E-AIM prediction of MSA equilibrium vapour pressure under conditions with free NH_3_ (3 times as many moles of NH_3_ as MSA). The dashed line is described by eqn (2) in the text. Above the dashed line, MSA is assumed not to condense; below the dashed line, MSA is assumed to condense. The data points from this study are presented as circles (without MSA condensation) and squares (with MSA condensation), colored by MSA concentration from 5 × 10^6^ to 2 × 10^8^ cm^−3^.

Another tool that has been used to estimate MSA volatility and condensation under different temperature and humidity conditions is the E-AIM, as presented by Hodshire *et al.*^67^Fig. 4 shows two volatility regimes of MSA as a function of RH and temperature based on E-AIM.^67^ In Fig. 4, the dashed line shows the threshold above which MSA is not expected to contribute to particle initial growth as it is expected to behave like a semi-volatile compound. Conversely, conditions below the dashed line indicate a regime where MSA is expected to condense onto particles and thus participate in particle growth. The model clearly shows that MSA is more likely to condense under higher RH and lower temperature conditions, which is qualitatively consistent with our experimental observations. The two circular data points indicate the experiments with no detectable MSA contribution to particle initial growth from 3.2 to 8 nm at approximately 16% RH at both −10 °C and +10 °C, which are in agreement with the model predictions. On the other hand, the four squares represent the experimental data points in which experiments show clear growth contribution from MSA. The experiments at −10 °C and −30 °C are consistent with E-AIM in showing condensation of MSA even though its absolute concentrations are relatively low compared to the experiments at dry conditions (16%). However, a notable discrepancy between E-AIM and the observations arises at +10 °C and 50% RH (bigger square one). Under these conditions, the E-AIM predicts no MSA condensation on particles, whereas experimental data clearly show that MSA condenses during the early stages of particle growth at rates approaching the kinetic limit. This inconsistency indicates that the effect of RH on MSA condensation is not adequately represented in the current E-AIM.

Although the E-AIM captures the general trend of increased MSA volatility with decreasing RH and increasing temperature, it underestimates MSA's capacity to condense under moderately humid and warm conditions, such as those typically found during the Arctic summer and in the marine boundary layer. Specifically, the combined experimental and modelling results point to the existence of a threshold regime in which MSA shifts from non-condensing to condensing behaviour. Our findings suggest that the effective condensation threshold of MSA lies between 16% and 50% RH, within the temperature range of −10 °C to +10 °C. This range likely defines the critical RH required for MSA-driven particle growth. To improve thermodynamic models, including E-AIM, further experiments are needed to better constrain the MSA volatility and refine it in models for predicting the volatility of MSA.

## 4 Conclusions and implications

New particle formation significantly affects the cloud condensation nuclei (CCN) concentrations, especially in pristine polar and marine atmospheres, thereby influencing cloud formation and ultimately climate. In this study, we conducted experiments in the CERN CLOUD chamber at different temperatures (+10 °C, −10 °C and −30 °C) across a wide range of RH (16–100%) to investigate the effect of RH on particle formation and initial growth in the MSA–H_2_SO_4_–H_2_O system under low levels of NH_3_, conditions typical for pristine environments. The results show that under dry conditions (16% RH), particle growth rates are relatively low and can be fully explained by sulfuric acid condensation. In contrast, under humid conditions (50% RH or higher), MSA makes a significant contribution to particle initial growth, as further confirmed by particle-phase composition measurements. The comparison to the nanoparticle growth model (MABNAG) reveals that the growth model cannot accurately reproduce the observed particle growth rates without accounting for MSA, except for the low RH cases where MSA does not contribute significantly to particle growth. At higher RH, particle growth rates are more accurately reproduced when MSA is treated as H_2_SO_4_ rather than through simplified parameterisations of MSA itself.

Furthermore, we directly compared the experimental data with E-AIM predictions of MSA volatility, using the same MSA properties as implemented in MABNAG. Although E-AIM catches the general behaviour of MSA condensation, predicting it to condense at lower temperatures and higher humidity, it is not consistent with all our experiments. Specifically, the model predicts no MSA condensation at +10 °C and 50% RH, whereas the experiments clearly show that MSA participates in early growth under those conditions.

These findings show that current model treatments of MSA are inadequate for predicting its participation in particle formation and early growth. In particular, MSA's contribution is likely underestimated in the warm and humid conditions of the marine boundary layer. Addressing this shortcoming requires targeted experiments to quantify the MSA-derived particle growth rates under varying conditions and refining the representation of MSA in particle growth processes. Such improvements are essential for advancing our understanding of new particle formation and early growth across a range of environments, especially in the free troposphere, where cold and dry conditions prevail, and in the marine boundary layer, where dimethylsulfide (DMS) is abundant.

## Author contributions

W. Y., R. B., J. S., J. D, L. C.-P., E. S., H. K., D. M. R., A. A., H. B.,N. B., V. B., A. C., R. C.-S., L. D., A. J., B. J., M. K. S., R. K., T. K., F. K., C. J. L., L. L., R. M., B. M., A. M., P. R., B. R., S. R., W. S., M. S., A. S., A. T., Y. T., J. T., N. S. U., J. W., B. Y., M. Z.-W., J. Z., Z. Z., J. C., I. E. H., R. F., H. H., A. K., T. P., R. V., P. M. W., M. K., N. M. D., J. K. and X.-C. H. prepared the CLOUD facility or measuring instruments. W. Y., R. B., J. S., J. D, L. C.-P., E. S., H. K., D. M. R., H. B., N. B., V. B., A. C., R. C.-S., L. D., A. J., B. J., M. K. S., R. K., T. K., F. K., C. J. L., L. L., B. M., A. M., P. R., S. R., W. S., M. S., A. S., Y. T., J. T., N. S. U., G. R. U., L. V., J. W., C. X., B. Y., M. Z.-W., J. Z., Z. Z., T. C., H. H., J. K. and X.-C. H. collected the data. W. Y., R. B., J. S., J. D, L. C.-P., E. S., H. K., D. M. R. and X.-C. H. analysed the experimental data. W. Y., A. H., T. Y.-J, I. R. and X.-C. H. carried out model simulation. W. Y., R. B., J. S., J. D, E. S., H. K., S. R., C. X., J. C., I. E. H., R. F., A. H., O. M., D. R. W., M. K., N. M. D., J. S., T. Y.-J., I. R., X.-C. H and K. L. contributed to the scientific discussion. W. Y., J. S., X.-C. H. and K. L. wrote the manuscript with the comments from R. B., J. D., E. S., H. K., N. B., F. K., S. R., G. R. U., C. X., J. C., I. E. H., R. F., A. H., A. K., N. M. D., J. K., T. Y.-J. and I. R.

## Conflicts of interest

At least one of the (co-)authors is a member of the editorial board of *Environmental Science: Atmospheres*. And the authors have no other competing interests to declare.

## Data Availability

Data for all figures in the main text are accessible at the zenodo repository (DOI: https://doi.org/10.5281/zenodo.18711242).
